# The REFLO-STEMI trial comparing intracoronary adenosine, sodium nitroprusside and standard therapy for the attenuation of infarct size and microvascular obstruction during primary percutaneous coronary intervention: study protocol for a randomised controlled trial

**DOI:** 10.1186/1745-6215-15-371

**Published:** 2014-09-25

**Authors:** Sheraz A Nazir, Jamal N Khan, Islam Z Mahmoud, John P Greenwood, Daniel J Blackman, Vijay Kunadian, Martin Been, Keith R Abrams, Robert Wilcox, AA Jennifer Adgey, Gerry P McCann, Anthony H Gershlick

**Affiliations:** Department of Cardiovascular Sciences, University of Leicester and the NIHR Leicester Cardiovascular Biomedical Research Unit, Glenfield Hospital, Groby Road, LE3 9QP Leicester, UK; Department of Cardiovascular Imaging, Division of Imaging Sciences & Biomedical Engineering, Rayne Institute, BHF Excellence Centre, St Thomas’ Hospital, King’s College London, London, UK; Multidisciplinary Cardiovascular Research Centre, Leeds Institute of Genetics, Health and Therapeutics, University of Leeds, Leeds, UK; Institute of Cellular Medicine, Faculty of Medical Sciences, Newcastle University and Cardiothoracic Centre, Freeman Hospital, Newcastle upon Tyne Hospitals NHS Foundation Trust, Newcastle upon Tyne, UK; Department of Cardiology, University Hospitals Coventry and Warwickshire NHS Trust, Coventry, UK; Centre for Biostatistics & Genetic Epidemiology, Department of Health Sciences, School of Medicine, University of Leicester, Leicester, UK; Faculty of Medicine & Health Sciences, Queen’s Medical Centre, Nottingham, UK; Heart Centre, Royal Victoria Hospital, Belfast, Northern Ireland, UK

**Keywords:** Cardiovascular magnetic resonance, Myocardial infarction, Microvascular obstruction, Adenosine, Nitroprusside, Primary angioplasty

## Abstract

**Background:**

Microvascular obstruction (MVO) secondary to ischaemic-reperfusion injury is an important but underappreciated determinant of short- and longer-term outcome following percutaneous coronary intervention (PCI) treatment of ST-elevation myocardial infarction (STEMI). Several small studies have demonstrated a reduction in the degree of MVO utilising a variety of vasoactive agents, with adenosine and sodium nitroprusside (SNP) being most evaluated. However, the evidence base remains weak as the trials have had variable endpoints, differing drug doses and delivery. As such, the results regarding benefit are conflicting.

**Methods:**

The REperfusion Facilitated by LOcal adjunctive therapy in STEMI (REFLO-STEMI) trial is a multicentre, prospective, randomised, controlled, open label, study with blinded endpoint analysis: Patients presenting within 6 h of onset of STEMI and undergoing planned primary PCI (P-PCI) with TIMI 0/1 flow in the infarct-related artery (IRA) and no significant bystander coronary artery disease on angiography, are randomised into one of three groups: PCI with adjunctive pharmacotherapy (intracoronary adenosine or SNP) or control (standard PCI). All receive Bivalirudin anticoagulation and thrombus aspiration. The primary outcome is infarct size (IS) (determined as a percentage of total left ventricular mass) measured by cardiac magnetic resonance imaging (CMRI) undertaken at 48 to 72 h post P-PCI. Secondary outcome measures include MVO (hypoenhancement within infarct core) on CMRI, angiographic markers of microvascular perfusion and MACE during 1-month follow-up. The study aims to recruit 240 patients (powered at 80% to detect a 5% absolute reduction in IS).

**Discussion:**

The REFLO-STEMI study has been designed to address the weaknesses of previous trials, which have collectively failed to demonstrate whether adjunctive pharmacotherapy with adenosine and/or SNP can reduce measures of myocardial injury (infarct size and MVO) and improve clinical outcome, despite good basic evidence that they have the potential to attenuate this process. The REFLO-STEMI study will be the most scientifically robust trial to date evaluating whether adjunctive therapy (intracoronary adenosine or SNP following thrombus aspiration) reduces CMRI measured IS and MVO in patients undergoing P-PCI within 6 h of onset of STEMI.

**Trial registration:**

Trial registered 20th November 2012: ClinicalTrials.gov Identifier NCT01747174.

**Electronic supplementary material:**

The online version of this article (doi:10.1186/1745-6215-15-371) contains supplementary material, which is available to authorized users.

## Background

Timely delivered primary percutaneous coronary intervention (P-PCI) has become the favoured reperfusion therapy for ST-elevation myocardial infarction (STEMI) in the US and Europe [1]. However, this interventional technique has not abolished the unpredictable phenomenon of no-reflow and the underappreciated, but potentially equally important, syndrome of normal epicardial-microvascular obstruction (MVO).

MVO describes abnormal tissue perfusion and/or coronary blood flow despite normal patency of the infarct-related artery (IRA) [2]. This can result in persistent myocardial injury and necrosis through interacting processes. Distal microembolisation of thrombus and plaque debris, activation of the inflammatory cascade, neutrophil plugging, toxic free-radical generation and capillary obstruction by intra-luminal (endothelial protrusion by cell swelling and cellular infiltrate rich in red-blood cells, platelets and granulocytes) and extra-luminal (compression from surrounding oedematous myocytes) mechanisms promote poor perfusion and irreversible injury to potentially viable myocytes [2–9]. These ultrastructural and functional changes result in a spectrum of MVO that, as detected by cardiac magnetic resonance imaging (CMRI), manifests in up to 70% of patients with STEMI treated with P-PCI [10–16]. Although the incidence of MVO varies between studies, presumably due to a combination of modifiable and non-modifiable patient-related factors, its presence has been reported to be associated with major adverse cardiac event (MACE) rates of up to 30% at 1 month and 60% at 12 months [11].

Manual thrombectomy has been shown to improve angiographic microvascular flow irrespective of the presence of visible thrombus [17], and to reduce infarct size (IS) and preserve microvascular integrity assessed by CMRI [18], leading to improved left ventricular (LV) function and tissue perfusion assessed by myocardial contrast echocardiography (MCE) [19]. However, there is conflicting evidence as to whether this leads to overall improved clinical outcomes [20–26] although the large ongoing TOTAL trial will provide further insight [27]. Glycoprotein IIb/IIIa (GPIIbIIIa) inhibitors further reduce IS and improve markers of microvascular perfusion in STEMI patients undergoing P-PCI [28–30]. Bivaluridin has been shown in the ACUITY [31] and HORIZONS-AMI [32] trials to provide similar efficacy with less bleeding and even reduced mortality compared with unfractionated heparin plus GPIIb/IIIa receptor inhibitors in high-risk patients undergoing PCI. However, residual mortality and subsequent MACE rates suggest there is room for improvement even in those patients who do not demonstrate slow or no-reflow angiographically.

Basic understanding of the MVO process has led to the evolution of several treatment regimens designed to improve outcomes, and include the use of vasodilators [33–41], albeit mostly in clinical trials. Of these, sodium nitroprusside (SNP) [12, 42–49] and adenosine [44, 50–62] and their effect on attenuating or preventing MVO have been the most studied. The randomised controlled trials of adenosine and SNP in P-PCI are presented in Table 1 (Additional file 1). Adenosine, aside from being a potent vasodilator [63], may have additional benefits due to its pleiotropic effects: the anti-inflammatory action of adenosine is well recognised [64, 65] and its ability to block the neutrophil-mediated processes that promote MVO may explain the reduction of reperfusion injury seen with intracoronary (IC) adenosine in canine infarct models [66]. Similarly SNP, a direct nitric oxide (NO) donor that requires no intracellular metabolism [67], utilises NO’s multiple vascular functions. These include vasodilatation of arterioles, inhibition of platelet adhesion and anti-inflammatory activity [68], which effectively reduce no-reflow in animal reperfusion-injury models [69, 70]. SNP and adenosine have, in some trials, demonstrated favourable improvement in electrocardiographic (ECG) and angiographic markers of microvascular perfusion, as well as improvements in short-term MACE [42, 44, 55, 71]. The randomised and placebo-controlled Acute Myocardial Infarction STudy of ADenosine (AMISTAD)-II trial sought to determine the benefit of adenosine in 2,118 patients presenting within 12 h of onset of anterior STEMI treated with thrombolysis (60%) or P-PCI (40%) [59]. IS and adverse clinical events were reduced in a sub-group who received a higher (70 μg/kg/min) dose of adenosine and in those reperfused within 3 h of symptom onset. This trial, although the largest to date, has a number of limitations in addition to the mixed reperfusion strategy cohort: (1) Adenosine was administered by intravenous (IV) infusion after the PCI; (2) IS was measured relatively late after presentation in only 11% of patients and by technetium-99 m sestamibi single-photon emission computed tomography (SPECT), which may underestimate IS compared to CMRI; and (3) no measure of myocardial salvage was obtained. Overall, AMISTAD-II appears not to be applicable in the modern P-PCI era.

**Table 1:** **Eligibility criteria**

Inclusion criteria	Exclusion criteria
● Aged ≥18 years	● Contraindications to: P-PCI, CMRI, gadolinium-based and/or iodinated contrast agents, or study medications: Adenosine, SNP, Aspirin, Thienopyridine and Bivalirudin
● Informed ASSENT (verbal consent) prior to angiography	
● STEMI ≤6 h of symptom onset, requiring P-PCI	
	● SBP ≤90 mmHg
● Single-vessel coronary artery disease (non-culprit disease <70% stenosis at angiography)	● Cardiogenic shock
	● Previous Q wave myocardial infarction
	● Culprit lesion not identified or located in a bypass graft
● TIMI flow 0/1 at angiography	
	● Stent thrombosis
● QTc <450 ms	● Left main disease
	● Known severe asthma
	● Known stage 4 or 5 chronic kidney disease (eGFR <30 mL/min/1.73 m^2^)
	● Pregnancy

CMRI, cardiac magnetic resonance imaging; e-GFR, estimated glomerular filtration rate; P-PCI, primary percutaneous coronary intervention; SBP, systolic blood pressure; TIMI, Thrombolysis in Myocardial Infarction.

The effects of adenosine on the coronary microcirculation during STEMI have only been assessed using CMRI in one previous study. Desmet et al. [51] assessed whether intracoronary administration of adenosine, distal to the occlusion site and immediately before initial balloon inflation, resulted in increased myocardial salvage and decreased MVO *versus* placebo on CMR at 48 to 72 h post P-PCI in 112 patients. They reported no significant difference in myocardial salvage between the two groups (41.3% vs. 47.8%, *P* = 0.52). MVO extent, angiographic markers of reperfusion and infarct size at 4 months were also similar in both groups. Interestingly, the authors reported a statistically significant benefit in favour of adenosine in patients with Thrombolysis in Myocardial Infarction (TIMI) 2-3 flow pre-PCI. This suggested that establishing flow prior to adenosine delivery was beneficial and perhaps necessary for the drug to have a clinical effect. As thrombectomy was not performed in this study, it is possible that adenosine may have been ineffective due to a combination of its short half-life and failure to reach the distal vascular bed. In addition, more patients had anterior MI in the adenosine group (48% vs. 33%). Anterior STEMI is known to be associated with larger ISs, reduced myocardial salvage and increased LV remodeling [72]. Moreover, the spontaneous reperfusion rate was high (28%) in this study, evident as TIMI 2-3 flow prior to P-PCI. The placebo group had almost twice as many patients with established TIMI 2-3 flow prior to PCI, and this is known to be associated with higher myocardial salvage and reduced IS. Finally, the expression of MVO indexed to the area at risk rather than IS or total LV mass has not been described previously in the evidence base and is unexplained in this study.

Although benefits have been shown for both adenosine and SNP in smaller trials, the results of such studies have been largely conflicting and hence, there is currently no consensus on the value of routine administration of adjunctive pharmaco-therapeutic agents to prevent or reduce MVO. In fact, a recent Cochrane review found that adenosine, when given as an adjunct during P-PCI, did not reduce all-cause mortality, non-fatal myocardial infarction or the incidence of angiographic no-reflow [73]. However, the authors conceded that the evidence base was limited and highlighted the need for further research with larger high quality trials. Heterogeneity in trial design (small numbers, sub-optimal drug dosages, inadequate anti-platelet therapy and variably chosen endpoints often lacking imaging confirmation of MVO and IS) has resulted in contradictory outcome data that may not be clinically applicable. Consequently there is divergent clinical practice, even within institutions. Furthermore, the incidence of no-reflow/MVO remains difficult to predict on coronary angiography alone. It could be argued that, given the strong relationship of MVO to prognosis, prophylactic prevention of MVO should be considered in all patients presenting with STEMI, irrespective of the thrombus burden, with delivery of agents theoretically able to reduce MVO.

The failure of some previous randomised clinical trials to show a reduction in MVO may be in part related to factors other than clinical efficacy. The lack of a sensitive imaging modality to detect MVO and failure to deliver vasoactive agents close to the microvascular bed may potentially have reduced their therapeutic impact.

We therefore designed the REperfusion Facilitated by LOcal adjunctive therapy in STEMI (REFLO-STEMI) study to evaluate whether adjunctive adenosine or SNP, administered in two doses (the first optimally delivered by distal intracoronary (IC) injection following thrombectomy), would be effective in preventing MVO and reducing IS, as determined with the sensitive measure of CMRI, in patients undergoing P-PCI for STEMI.

## Methods

**Figure 1:**
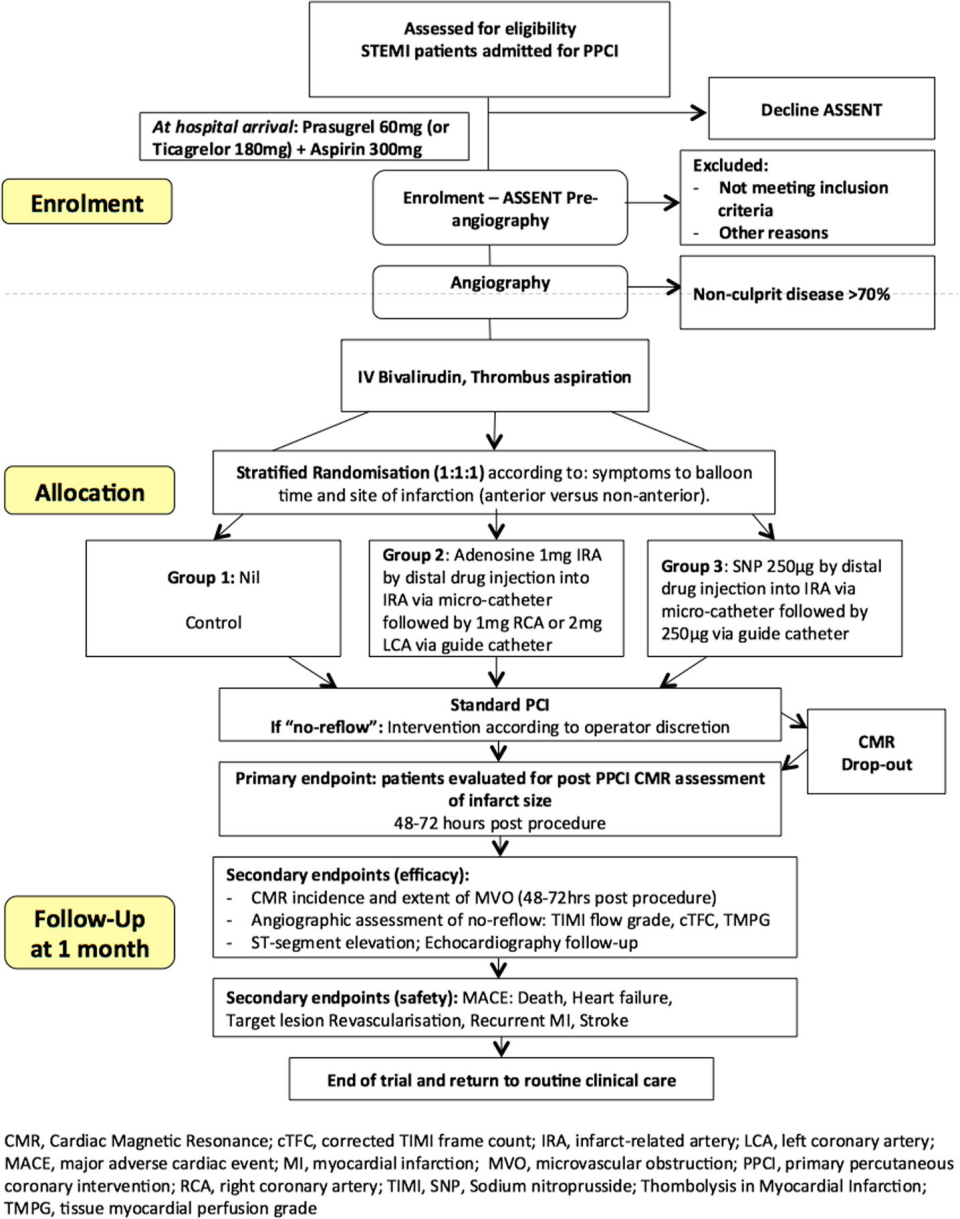
**Study recruitment diagram.**

The REFLO-STEMI trial is a multicentre, randomised, controlled, open label, clinical trial (see Figure 1) in four regional cardiac centres in the United Kingdom, conducted in compliance with the principles of the Helsinki Declaration. Ethical approval for the study (reference 11/H0405/10) was obtained from the National Research Ethics Service (UK). All patients presenting within 6 h of symptom onset of STEMI, who are suitable for reperfusion by P-PCI and have a baseline corrected QT interval (QTc) <450 ms on admission ECG (to limit the risk from the possible QT prolongation effect of the study drugs), are provisionally eligible to participate in the study. TIMI flow grade 0-1 in the IRA and no flow-limiting bystander disease (that is, no stenosis ≥70% in non-infarct-related arteries (N-IRA)) are pre-requisites to randomisation (see Table 1 for detailed eligibility criteria). Following verbal consent or assent [74, 75] patients will be randomised 1:1:1 to: adjunctive IC adenosine, SNP or control (standard P-PCI alone) using a dedicated 24/7 computerised telephone service (provided by the ‘Sealed Envelope Company’, UK) with three stratifications: 1, ‘symptoms to balloon <3 h or ≥3 h’; 2, ‘anterior infarction’ or not; and 3, recruiting centre.

In all cases, P-PCI will be performed in line with accepted practice with trans-radial or femoral arterial access using 6-7 Fr sheaths. Patients will be pre-treated with dual antiplatelet therapy with aspirin (300 mg loading dose and 75 mg/day maintenance) and Prasugrel (60 mg loading dose and 10 mg/day maintenance) [76, 77] or Ticagrelor (loading dose 180 mg and maintenance dose of 90 mg twice daily) and given for up to 12 months [78–80]. Bivalirudin will be administered to all patients (0.75 mg/Kg bolus plus infusion of 1.75 mg/Kg/hr) in the absence of specific contraindication, with dose reduction for renal insufficiency, and will be discontinued at the completion of P-PCI (but could be continued for 4 h if clinically indicated). For patients randomised to an intervention arm, following manual thrombectomy and thorough flushing of the catheter, the first drug dose (adenosine 1 mg or SNP 250 mcg) will be injected as distally as possible via the thrombus aspiration catheter. Immediately following stent deployment, providing repeat measure of QTc is <450 ms and remains <60 ms increase over baseline value, the second drug dose (adenosine 1 mg if IRA is the right coronary artery (RCA) otherwise 2 mg or SNP 250 mcg) will be injected via the guide catheter. Administering the second drug dose distal to the stent was considered but the risk associated with crossing the stent with the thrombectomy catheter was thought to outweigh the benefit of distal drug delivery. The ECG will be recorded and retained at each angiography time point. The degree of ST-segment resolution (STR) will be determined from 12-lead ECGs acquired pre- and post-P-PCI and categorised as complete (>70%), partial (30% to 70%), or no (<30%) STR [16, 81]. The maximal sum of ST-segment elevation, measured 60 ms after the J point, will be calculated from three contiguous leads in the infarct territory. Angiographic images will be acquired at 30 frames per second with long acquisitions (to visualise the venous phase in contrast passage) in orthogonal views before intervention and after stenting (at the time of the final/optimal angiographic result) to enable determination of angiographic markers of MVO offline at a core laboratory (Newcastle University). TIMI myocardial perfusion grade (TMPG) will be assessed visually as previously described [82, 83] (Additional file 1). Digital quantification of myocardial perfusion or ‘blush’ will be performed using ‘QuBE’ software [84]; corrected TIMI frame count (cTFC) will be calculated as the number of cine-frames needed for dye to reach standardised distal landmarks, to objectively evaluate coronary blood flow as a continuous variable [85, 86]. A list of angiographic markers of MVO to be assessed is provided in Table 2.

**Table 2:** **Study outcome measures**

Type of outcome measure	Outcome measures
CMRI parameters	● IS (% total LV mass): Primary outcome
	● Incidence and extent of MVO (% LV mass)
	● Myocardial salvage index (MSI)
	● Intra-myocardial haemorrhage (IMH)
	● LV ejection fraction (LVEF) and volumes
Angiographic markers of MVO	● TIMI flow grade [87]
	● Corrected TIMI frame count (cTFC) [85, 86]
	● TIMI myocardial perfusion grade (TMPG) [82, 83, 88]
	● Computer-assisted myocardial blush quantification using the software ‘Quantitative Blush Evaluator’ (QuBE) [84]
	● Incidence pre- and post-procedure of angiographic true ‘no-reflow’
	● Incidence of angiographic slow/no-reflow after P-PCI
ECG	● Degree of ST segment resolution on ECG [16, 81]
Echocardiography	● LV function at baseline and 3 months
Sub-analyses	● Comparing CMRI markers with other myocardial perfusion markers (angiographic, ECG and cardiac enzymes)
	● Overall MACE and its components at 1 month: death, need for TLR, recurrent MI, severe heart failure and CVE

CMRI, cardiac magnetic resonance imaging; CVE, cerebrovascular event; ECG, electrocardiogram; e-GFR, estimated glomerular filtration rate; LV, left ventricular; LVEF, MACE, major adverse cardiac events; MI, myocardial infarction; MVO, microvascular obstruction; P-PCI, primary percutaneous coronary intervention; SBP, systolic blood pressure; TIMI, Thrombolysis in Myocardial Infarction; TLR, target lesion revascularisation.

Following the P-PCI procedure, and when clinically stable, the patient will be provided with a detailed study information leaflet and written informed consent will be obtained from each participant to continue partaking in the trial. A 20% drop out rate between P-PCI and CMR has been allowed for. Studies on informed consent in acute MI patients have suggested that oral information is far better received, processed and recalled by patients compared with the written form [89, 90]. In the ISIS-4 patient cohort, 95% recalled receiving the oral information, whereas only 37% recalled receiving the written consent form [89]. Furthermore, only 18% of 346 patients prospectively studied reported reading the patient information sheet before providing or refusing consent to participate in the HERO-2 acute MI trial [90]. Of particular note is that patients who gave consent were more likely to report good or partial understanding of the written material than those who refused consent. This raises the possibility of selection bias at the time of consent. Consequently, we believe verbal explanation of a trial may be a more effective and valuable source of information than a written consent form in the emergent situation of STEMI, where treatment must be provided without undue delay. This approach has been successfully used in two recent STEMI trials [74, 75].

Blood samples will be drawn at baseline and at 4, 12 and 24 h after P-PCI for cardiac enzymes (CK-MB and Troponin) estimation and at pre-discharge for NT-proBNP. ECG recording will be undertaken at 90 min, 24 h and pre-discharge. All patients will be commenced on a beta-blocker, angiotensin converting enzyme (ACE) inhibitor and high-dose statin in addition to dual antiplatelet therapy, unless contra-indicated, according to international guidelines.

**Figure 2:**
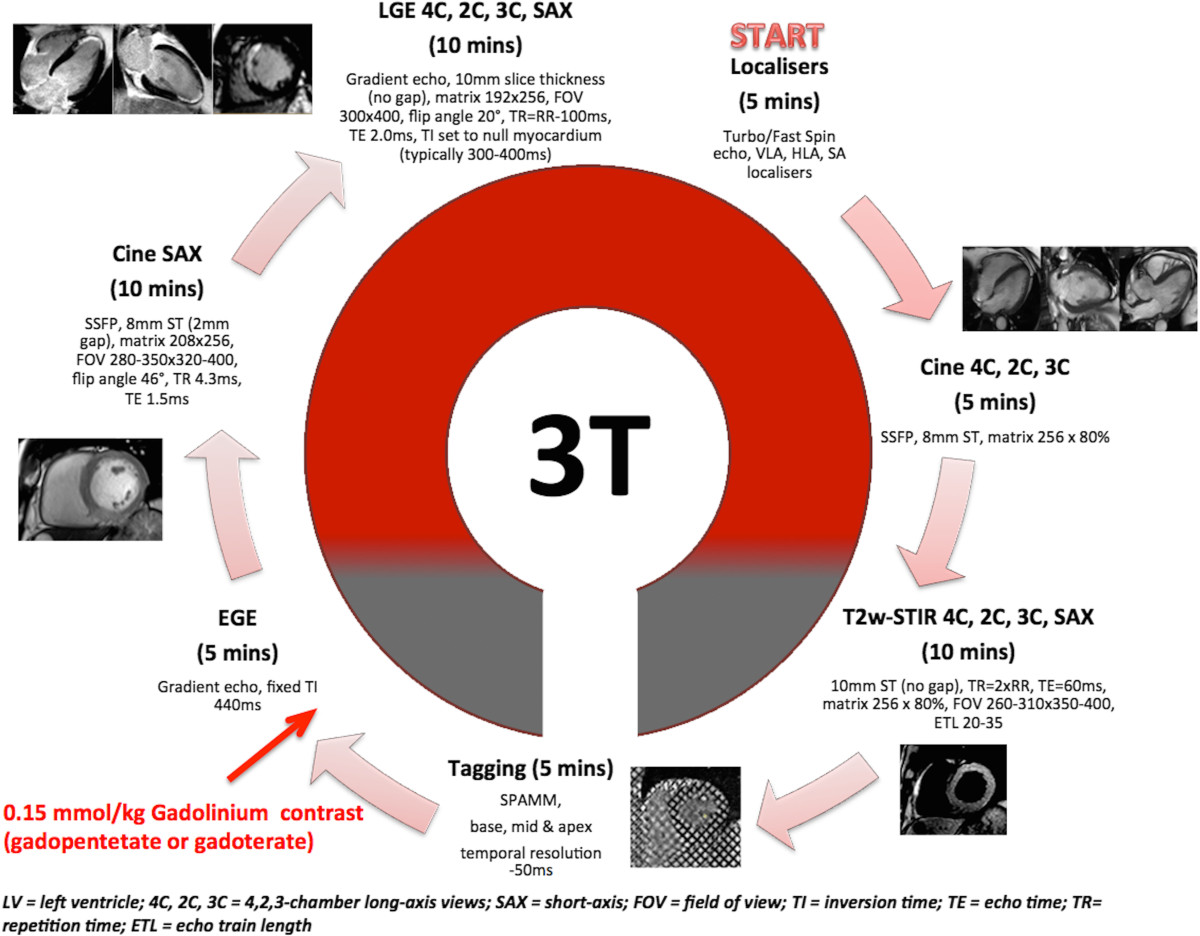
**CMRI protocol.**

Patients will undergo CMRI at 48 to 72 h after presentation with STEMI on a 3.0 T scanner with retrospective electrocardiographic gating and dedicated cardiac receiver coils at each of the four participating centres (see Figure 2) to provide the primary endpoint [91, 92]. Prior to contrast administration, T2-weighted short-tau inversion recovery (T2w-STIR) imaging with coil SI correction will be performed in long-axis (LAX) views and contiguous short-axis (SAX) slices covering the entire LV to assess for oedema (area at risk, (AAR)). Three SAX (base, mid and apical) tagged images will be acquired using a prospectively gated spatial modulation of magnetization (SPAMM) gradient-echo sequence. Early gadolinium enhancement (EGE) imaging will be acquired 1 to 3 min after 0.15 mmol/kg gadolinium-DTPA (Magnevist, Bayer, Germany) administration using a single-shot inversion-recovery gradient-echo sequence. Functional assessment of LV ejection fraction (LVEF), volumes and mass will be according to current standards with the use of a steady state free precession (SSFP) cine pulse sequence covering the whole LV with 8 to 12 contiguous short axis (SAX) slices. Late gadolinium enhancement (LGE) imaging [93] will then be performed in LAX (2-, 3- and 4-chamber) views and contiguous SAX slices covering the whole LV. LGE images will be acquired 10 to 15 min post contrast using a segmented inversion-recovery gradient-echo sequence. The inversion time will be progressively adjusted to null unaffected myocardium. Study outcome measures are listed in Table 3.

**Table 3:** **Definitions of adverse events**

Adverse event	Definition
Cardiogenic shock	Systolic blood pressure <90 mmHg for at least 30 min (or the need for supportive measures to maintain a systolic blood pressure of >90 mmHg) in the presence of a heart rate of >60 beat/min in association with signs of end-organ hypoperfusion (cold extremities, low urinary output <30 mL/h and/or mental confusion)
Myocardial infarction (MI)	MI will be defined differently in specific clinical situations in this trial. The European Society of Cardiology (ESC) and American College of Cardiology (ACC) criteria for acute, evolving or recent MI will apply
Re-infarction	Further chest pain during the index admission lasting >20 min accompanied by new electrocardiographic changes (new Q waves >0.04 s or ST-segment elevation >0.1 mV in two leads for >30 min), further enzyme rise or both
Recurrent MI	A ≥20% rise in the value of the biomarker measured serially 6 to 12 h apart, provided the absolute value is greater than the 99% percentile upper reference limit. For patients who die and for whom no cardiac markers were obtained, the presence of new ST segment elevation and new chest pain would meet criteria for MI
Contrast-induced nephropathy	25% increase in serum creatinine concentration from the baseline value, or absolute increase of at least 0.5 mg/dL (44.2 μmol/L), appearing within 48 h of administration of contrast media, and maintained for 2 to 5 days [94–96]
Cerebrovascular events	Stroke is defined as a new focal neurological deficit of presumed vascular aetiology persisting >24 h combined with a neurological imaging study that does not indicate a different aetiology. Transient ischaemic attack (TIA) is any focal ischaemic neurological deficit of abrupt onset, which resolves completely within 24 h
Severe heart failure	Early heart failure: any new onset cardiogenic shock or heart failure occurring after randomisation and during the index admission with radiographic evidence of pulmonary oedema requiring intravenous diuretic therapy
	Late heart failure: admission to hospital for treatment for documented New York Heart Association (NYHA) class III or IV heart failure
Major bleeding	Defined according to the TIMI criteria as fatal bleeding, any intracranial bleeding or clinically overt signs of haemorrhage associated with a drop in haemoglobin (Hb) of ≥ 50 g/L

CMRI analysis, blinded to patient details, will be undertaken in a central core lab (University of Leicester) using cmr42 (Circle Cardiovascular Imaging, Calgary, Canada). Anonymised CMR images will be graded for image quality using a 4 point scale before analysis: 4 = excellent; 3 = good; 2 = moderate; and 1 = non-analysable. Endocardial and epicardial borders will be manually contoured on contiguous SAX LV slices, excluding papillary muscles, trabeculae and blood-pool artefact for LV volumetric, AAR and IS analyses. Infarct will be identified as enhancement on LGE images and quantified using the Full-Width Half-Maximum (FWHM) technique [97]. MVO will be defined (and quantified) as hypoenhancement within infarcted myocardium, as determined from LGE images, and will be included in the total IS. Myocardial oedema will be quantified using semi-automatic thresholding defining AAR as enhancement within myocardium of signal intensity >2 standard deviations (SD) above that of a region of interest (ROI) contoured in remote myocardium. Hypoenhanced areas within the AAR will be regarded as intra-myocardial haemorrhage (IMH). Myocardial salvage index (MSI) will be calculated as: 100*((AAR-IS)/AAR). IS, MVO, AAR and IMH will be expressed as a percentage of LV end-diastolic mass (%LVM) and LV volumes will be indexed to body-surface area. Intra- and inter-observer variability will be reported for the primary outcome measure.

All patients will be followed up for at least 1 month following randomisation and throughout the course of the study until the last patient recruited to the trial has completed 1-month follow-up. Median follow-up will be reported. Patients will also be flagged with the Office for National Statistics to ensure mortality data are captured. It is anticipated that most adverse events will be expected as recognised complications of STEMI or the revascularisation procedure. Such events will be recorded for the evaluation of outcome measures and for safety monitoring. Definitions of important adverse events are provided in Table 3. Investigators will be required to notify the coordinating centre (University Hospitals of Leicester, UK) within 24 h if any of the following adverse events occur: death; a serious deterioration in a patient’s health that results in life-threatening injury or illness; an event resulting in permanent impairment of a body structure or function; an event resulting in medical or surgical intervention to prevent permanent impairment to body structure or function; an event prolonging inpatient hospitalisation. On receipt of notification of any trial adverse or clinical event, the co-coordinating centre will request additional details specific to the nature of the event and carefully monitor these episodes overall. A clinical events committee has been established to review and adjudicate key trial adverse events, blinded to patient details and treatment allocation, using original source documents.

### Statistical methods

Demographics will be presented and values of IS and MVO will be summarised, both overall and by treatment group. The distribution of IS will be investigated and the data will be transformed if found to be non-normally distributed. Primary analysis will be by intention to treat with a secondary analysis by treatment received. Patients entering into the study but not completing the CMRI will continue to be followed-up for MACE on an intention-to-treat basis. Analysis of Variance (ANOVA) will compare mean IS between groups. Each drug will be compared to the control (that is, Adenosine *vs.* Control and SNP *vs.* Control). Multivariable analysis using linear regression will take into consideration possible confounders such as sex, age and other co-morbidities. The major confounders of location of infarct (anterior/non-anterior) and time from symptom onset to reperfusion will be addressed by the stratified randomisation process. Other important confounders, such as collateral blood flow to the infarct territory determined by the Rentrop score [98], will be controlled for in the statistical analysis. Secondary endpoint analysis will employ time-to-event regression methods to investigate potentially important predictors of MACE.

### Sample size

Sample size has been based on previous observations of significant correlation between the extent of CMR measured MVO and IS (which on average is 20% of LV mass as detected by CMR after P-PCI) [16]. Since there are no available data regarding the incidence of MVO with the study drugs assessed by CMR, and the wealth of published data on IS following P-PCI, we have chosen IS as the primary endpoint of the trial. IS is a powerful predictor of ventricular function, adverse LV remodeling and short-medium term clinical outcome [13, 15, 16, 99–111]. Furthermore, new infarct size of 4% of LV mass has been shown to be associated with adverse prognosis in patients with coronary artery disease undergoing revascularisation-related injury [112]. To detect a reduction in IS from 20% to 15% of LV mass, assuming a standard deviation of 10% [18, 102, 108, 109, 113–117], α of 0.05, two-tailed, 80% power and a drop-out rate of 20% between P-PCI and CMR, 80 subjects per group (240 in total) will be required.

### Study organisation

The study is funded by the Medical Research Council (MRC) and managed by the National Institute for Health Research (NIHR) on behalf of the MRC-NIHR partnership. The trial sponsor is the University Hospitals of Leicester NHS Trust. Trial support will be provided by the Leicester Clinical Trials Unit (UK Clinical Research Collaboration (UKCRC) ID 43) who will be responsible for database provision, data management and statistical analysis. The study will be overseen by a Trial Steering Committee (TSC), with an independent chair and two additional independent members, which will have access to the database after study completion and data-lock. Efficacy and safety data (particularly unexpected adverse events) will be scrutinised by an Independent Data and Safety Monitoring Board (DSMB), which will report back to the TSC. Clinical trials number http://NCT01747174 Clinicaltrials.gov.

## Discussion

The REFLO-STEMI study has been designed to address the weaknesses of previous trials, which have collectively failed to demonstrate whether adjunctive pharmacotherapy with adenosine and/or SNP can reduce measures of myocardial injury (infarct size and MVO) and improve clinical outcome, despite good basic evidence that they have the potential to attenuate this process. The REFLO-STEMI trial will be the first study to combine what are considered appropriate efficacious drug dosages, delivered optimally to the site of maximal benefit, with the use of CMR to robustly measure reperfusion success, in a group of patients treated with a contemporary reperfusion strategy. The study will be powered accordingly to deliver a definitive answer as to whether these agents can reduce infarct size. Additional measures of myocardial perfusion (angiographic and electrocardiographic) and early clinical outcome data will provide further insight in to the potential role of prophylactic adjunctive pharmacotherapy, administered universally for STEMI patients or for those selected by retrospective analyses to most benefit, augmenting the benefits of timely-delivered P-PCI. As the largest and most scientifically robust trial to date, the REFLO-STEMI study, alongside the existing combination of studies, will inform future STEMI Guideline committees.

## Trial status

The REFLO-STEMI trial has successfully completed recruitment of 247 patients. Follow-up and data collection are in progress and all investigators remain blinded to outcome data.

## Electronic supplementary material

Additional file 1: Table S1: Main randomised controlled trials investigating the role of adenosine and sodium nitroprusside (SNP) in attenuating or preventing MVO in STEMI treated with P-PCI [42, 44, 45, 51–53, 55–57, 60, 61, 118, 119]. **Table S2**. TIMI myocardial perfusion grade (TMPG) [82]. **Table S3**. TIMI flow grade (TFG) classification [87]. (DOC 62 KB)

Below are the links to the authors’ original submitted files for images.

Authors’ original file for figure 1

Authors’ original file for figure 2

## Abbreviations

AAR: Area at risk
ACC: American College of Cardiology
ACE: Angiotensin converting enzyme
ANOVA: Analysis of variance
CHF: Congestive heart failure
CK-MB: Creatine kinase MB isoenzyme
CMRI: Cardiac magnetic resonance imaging
CS: Cardiogenic shock
cTFC: Corrected TIMI frame count
CVD: Cardiovascular death
CVE: Cerebrovascular event
DSMB: Data and Safety Monitoring Board
ECG: Electrocardiogram
ESC: European Society of Cardiology
EGE: Early gadolinium enhancement
eGFR: Estimated glomerular filtration rate
FWHM: Full width half maximum
GPIIbIIIa: Glyocprotein IIbIIIa
Hs-CRP: High sensitivity C-reactive protein
IRA: Infarct-related artery
IC: Intra-coronary
IMH: Intra-myocardial haemorrhage
IS: Infarct size
LAX: Long axis
LBBB: Left bundle branch block
LCA: Left coronary artery
LGE: Late gadolinium enhancement
LV: Left ventricular
LVEDV: LV end-diastolic volume
LVEF: LV ejection fraction
LVM: LV end-diastolic mass
MACE: Major adverse cardiac events
MBG: Myocardial blush grade
MBV: Myocardial blood volume
MCE: Myocardial contrast echocardiography
MRC: Medical Research Council
MSI: Myocardial salvage index
MVD: Multi vessel disease
MVO: Microvascular obstruction
NIHR: National Institute for Health Research
N-IRA: Non-infarct related artery
NR: No-reflow
NT-proBNP: N-terminal pro-brain natriuretic peptide
NYHA: New York Heart Association
OS: Observational study
P-PCI: Primary percutaneous coronary intervention
RCA: Right coronary artery
ROI: Region of interest
SAX: Short axis
SBP: Systolic blood pressure
SD: Standard deviations
SNP: Sodium nitroprusside
SPAMM: Spatial modulation of magnetisation
SSFP: Steady state free precession
STEMI: ST-elevation myocardial infarction
STD: ST-segment deviation
STR: ST-segment resolution
SPECT: Sestamibi single-photon emission computed tomography
T2W: T2-weighted
T2w-STIR: T2W short tau inversion recovery
TA: Thrombus aspiration
TIA: Transient ischaemic attack
TIMI: Thrombolysis in Myocardial Infarction
TFG: TIMI flow grade
TLR: Target lesion revascularisation
TMPG: TIMI myocardial perfusion grade
TSC: Trial Steering Committee
TVR: Target vessel revascularisation
UKCRC: United Kingdom Clinical Research Collaboration.
