# Supplementary material for: The REFLO-STEMI trial comparing intracoronary adenosine, sodium nitroprusside and standard therapy for the attenuation of infarct size and microvascular obstruction during primary percutaneous coronary intervention: study protocol for a randomised controlled trial
Source: Trials. 2014 Sep 25;15:371. doi: 10.1186/1745-6215-15-371 (PMC4189551; doi:10.1186/1745-6215-15-371)
Supplement: Supplementary file 1 — Additional file 1: Table S1: Main randomised controlled trials investigating the role of adenosine and sodium nitroprusside (SNP) in attenuating or preventing MVO in STEMI treated with P-PCI [42, 44, 45, 51–53, 55–57, 60, 61, 118, 119]. Table S2. TIMI myocardial perfusion grade (TMPG) [82]. Table S3. TIMI flow grade (TFG) classification [87]. (DOC 62 KB) [file 13063_2014_2242_MOESM1_ESM.doc]

Additional file

Table S1. Main randomised controlled trials investigating the role of adenosine and sodium nitroprusside (SNP) in attenuating or preventing MVO in STEMI treated with P-PCI.

| Study/Author | Year | Main inclusion / exclusion | No. of patients (study drug/n) | Age (years) and time to P-PCI (min) | Dose administered | Mode of drug delivery | Primary outcome(s) | Secondary outcome(s) | Main findings | Main limitations |
| --- | --- | --- | --- | --- | --- | --- | --- | --- | --- | --- |
| Adenosine | | | | | | | | | | |
| REOPEN-AMI [44] | 2013 | <12 h symptoms; TFG 0-1 flow in IRA pre-PCI; ‘Rescue’ PCI; Excluded LBBB | 80/240 | 63; 278 | 120 μg rapid bolus then 2 mg over 2 min | Selective IC | STR >70% | TFG; MBG; 30d MACE | ↑ STR; ↑ MBG; ↓ TFC | No direct assessment of IS/MVO |
| Zhang et al. [61] | 2012 | <12 h symptoms; ‘new’ LBBB; TIMI 0–3; excluded MVD | 59/90 | 63; 279 | 50 or 70 μg/kg/min | IV infusion (3 h) | IS (SPECT) | TFG; MBG; Incidence NR; LV function; MACE at 6 m | ↓ IS (at 6 m); ↑ MBG; ↓ NR | No CMRI - IS may be underestimated by SPECT; IS only in 60%; MSI not assessed; no reported use of TA or GPIIbIIIa inhibitors |
| Desmet et al. [51] | 2011 | <12 h symptoms; ‘new’ LBBB; TFG 0-3 | 56/110 | 61; *215 (150, 296) and 193 (150, 305) for adenosine and placebo, respectively | 4 mg | Selective IC | MSI and MVO (CMRI) | TFG; TFC; MBG; STR; 30d and 1 year MACE | No benefit | No routine TA; No details on IS data; assessed ‘early’ rather than ‘late’ MVO; no stratified randomisation: more anterior MIs in adenosine arm and ↑ (almost double) spontaneous reperfusion rate in control arm |
| Grygier et al. [53] | 2011 | <6 h symptoms; TFG 0-2 | 35/70 | 65; 249 | LCA: 2 mg; RCA: 1 mg | IC via guide catheter | MBG; STR | TFG; TFC; 30d MACE | ↑ MBG; ↑ STR; ↓ MACE | No imaging assessment of IS; No routine TA |
| Fokemma et al. [52] | 2009 | <12 h symptoms; TFG 0-3; included MVD | 226/448 | 62; *180 (130, 288) and 165 (125, 255) adenosine *vs.* placebo | 2 x 120 μg | IC via guide catheter | Residual STD | STR; MBG, TFG; IS (enyzymatic); 30d MACE | No benefit | No imaging assessment of IS; low drug dose injected proximally via guide catheter |
| Stoel et al. [60] | 2008 | STR <70% and persistent ST↑ post-P-PCI with TFG 2-3 and <30% residual stenosis | 27/49 | 67; 220 | 60 mg | Selective IC (over 5-10 min) | STR | TFC; MBG; 1 year MACE | ↑ STR; ↑ MBG; ↓ TFC | Pre-selected patients with sub-optimal reperfusion success; no imaging assessment of IS; no TA; shorter ischaemic time in adenosine arm; no ITT reported |
| Micari et al. [56] | 2005 | 6 h symptoms; excluded if previous MI | 14/30 | 57; 292 | 50 or 70 μg/kg/min | IV infusion (3 h) | MSI | MBV in AAR | ↑ MSI, ↑ MBV | IS and AAR assessed by MCE; no TA; low study numbers; used anterior MI patients from AMISTAD-II |
| Petronio et al. [57] | 2005 | <6 h symptoms; TFG 0-1; included MVD | 30/90 | 59; 179 | 4 mg | Selective IC | LV remodelling (LVEDV ≥20% on echo) at 6 months | Angiographic NR; TFC; ΔLVEDV | Improved angiographic results but no ↓ prevalence of remodelling | No direct IS assessment; selection bias (‘alternate’ randomisation); allowed use of other vasoactive drugs if NR occurred |
| Marzilli et al. [55] | 2000 | <3 h symptoms; TFG 0-2; included MVD | 27/54 | 60; 116 | 4 mg | Selective IC | Feasibility; safety; TFG | LV function; in-hospital clinical events | Well tolerated, feasible; ↑ flow, ↑ ventricular function, ↓ incidence NR | As for ATTACC; no outpatient follow-up |
| SNP | | | | | | | | | | |
| Zhao et al. [118] | 2013 | <12 h symptoms; TFG 0-2; age ≤75 years; | 80/162 | 63; 345 | 100 μg rapid bolus | Selective IC | TFC; MBG; STR >70% | TFG; LVEF (echo); MACE at 6 m | ↓ TFC; ↑ STR; ↑ MBG; ↑ LVEF | No imaging assessment of IS |
| REOPEN-AMI [44] | 2013 | <12 h symptoms; TFG 0–1; ‘Rescue’ PCI; excluded LBBB | 80/240 | 63; 278 | 60 μg rapid bolus then 100 μg over 2 min | Selective IC | STR >70% | TFG; MBG; 30d MACE | No benefit | See above; low dose SNP; more anterior MIs in SNP arm; ECG surrogate for IS |
| Pan et al. [119] | 2009 | <12 h symptoms; TFG 0-3 | 46/92 | 53; 492 | 100 μg bolus; repeated after 5 min if TFG <3 | Selective IC | TFG; TFC; MCE parameters | Plasma Hs-CRP and NT-proBNP; MACE at 6 m | ↑ TFG; ↓ TFC; ↓ MACE; improved MCE parameters | No direct imaging assessment of IS/MSI; TA at operator discretion (63%) |
| Parikh et al. [45] | 2007 | High-risk ACS; TIMI 0-2 | 26/75 | 55; N/A | 50 μg/bolus SNP ± 12 μg/bolus Adenosine | IC via guide catheter | TFG; MBG (visual) | MACE at 6 m | Combination of adenosine + SNP ↑ TFG, ↑ MBG and ↓ MACE cf Adenosine alone | Included non-STEMI ACS patients; no ‘SNP only’ arm; fewer high-risk lesions and greater prevalence of triple vessel disease (possible pre-conditioning effect) in combination arm |
| Amit et al. [42] | 2006 | <12 h symptoms; TFG 0-2; excluded LBBB | 48/98 | 62; *202 (146, 311) and 240 (146, 325) for SNP and placebo, respectively | 60 μg | Selective IC | TFC; STR >70% | TFG; MBG; MACE (TLR, MI or death) at 6 m | No ↓ TFC or MBG but ↓ MACE | No IS assessment; underpowered; no TA; only 45% received GPIIbIIIa inhibitor |

AAR, area at risk; CHF, congestive heart failure; CMRI, cardiac magnetic resonance imaging; CS, cardiogenic shock; CVD, cardiovascular death; GPIIbIIIa, glycoprotein IIbIIIa; Hs-CRP, high sensitivity C-reactive protein; IC, intracoronary; IS, infarct size;, ITT, intention to treat; IV, intravenous; LBBB, left bundle branch block; LCA, left coronary artery; LV, left ventricular; LVEDV, LV end-diastolic volume; MACE, major adverse cardiac events; MBV, microvascular blood volume; MCE, myocardial contrast echocardiography; MI, myocardial infarction; MSI, myocardial salvage index; MBG, myocardial blush grade; MVD, multi-vessel disease; NR, no-reflow; NT-proBNP, N-terminal prohormone brain natriuretic peptide; OS, observational study; P-PCI, primary percutaneous coronary intervention; RCA, right coronary artery; SPECT, single-photon emission computed tomography; STD, ST-segment deviation; STEMI, ST-elevation MI; STR, ST-segment resolution (defined as >50% unless specifically stated); TA, thrombus aspiration; TIMI, Thrombolysis in Myocardial Infarction; TFC, TIMI frame count; TFG, TIMI flow grade; TLR, target lesion revascularisations; TVR, target vessel revascularisation.

*Median and interquartile range reported.

Table S2. TIMI myocardial perfusion grade (TMPG) [82]

| Grade of myocardial perfusion | Definition |
| --- | --- |
| TMPG 0 | Failure of dye to enter the microvasculature |
| TMPG 1 | Dye slowly enters but fails to exit the microvasculature |
| TMPG 2 | Delayed entry and exit of dye from the microvasculature |
| TMPG 3 | Normal entry and exit of dye from the microvasculature |

Table S3. TIMI flow grade (TFG) classification [87]

| TFG | Definition |
| --- | --- |
| Grade 0 | No perfusion |
| Grade 1 | Penetration without perfusion |
| Grade 2 | Partial perfusion |
| Grade 3 | Complete perfusion |
