# Supplementary figures and images for: The REFLO-STEMI trial comparing intracoronary adenosine, sodium nitroprusside and standard therapy for the attenuation of infarct size and microvascular obstruction during primary percutaneous coronary intervention: study protocol for a randomised controlled trial
Source: Trials. 2014 Sep 25;15:371. doi: 10.1186/1745-6215-15-371 (PMC4189551; doi:10.1186/1745-6215-15-371)

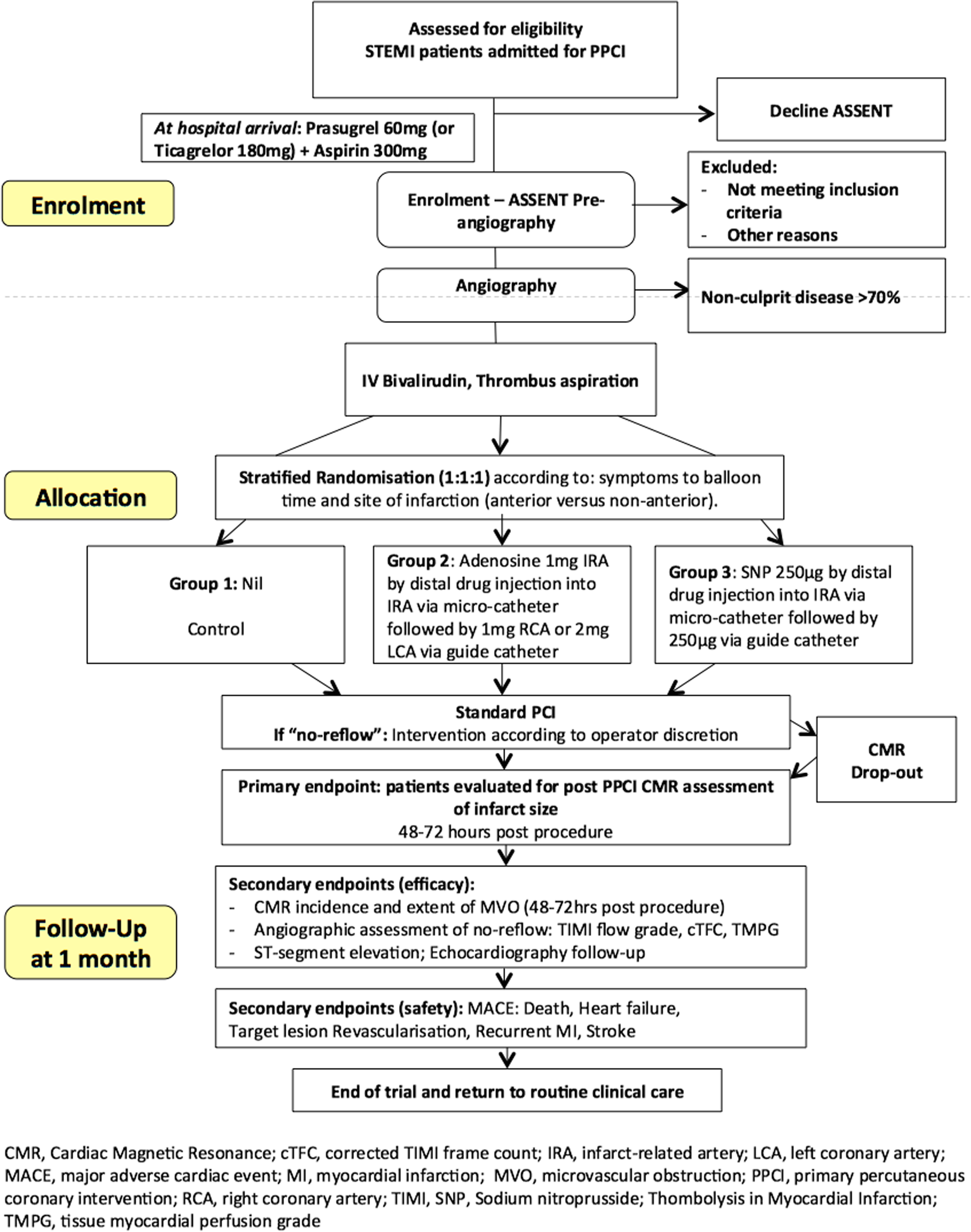

Supplement: Supplementary file 2 — Authors’ original file for figure 1 [file 13063_2014_2242_MOESM2_ESM.tif]

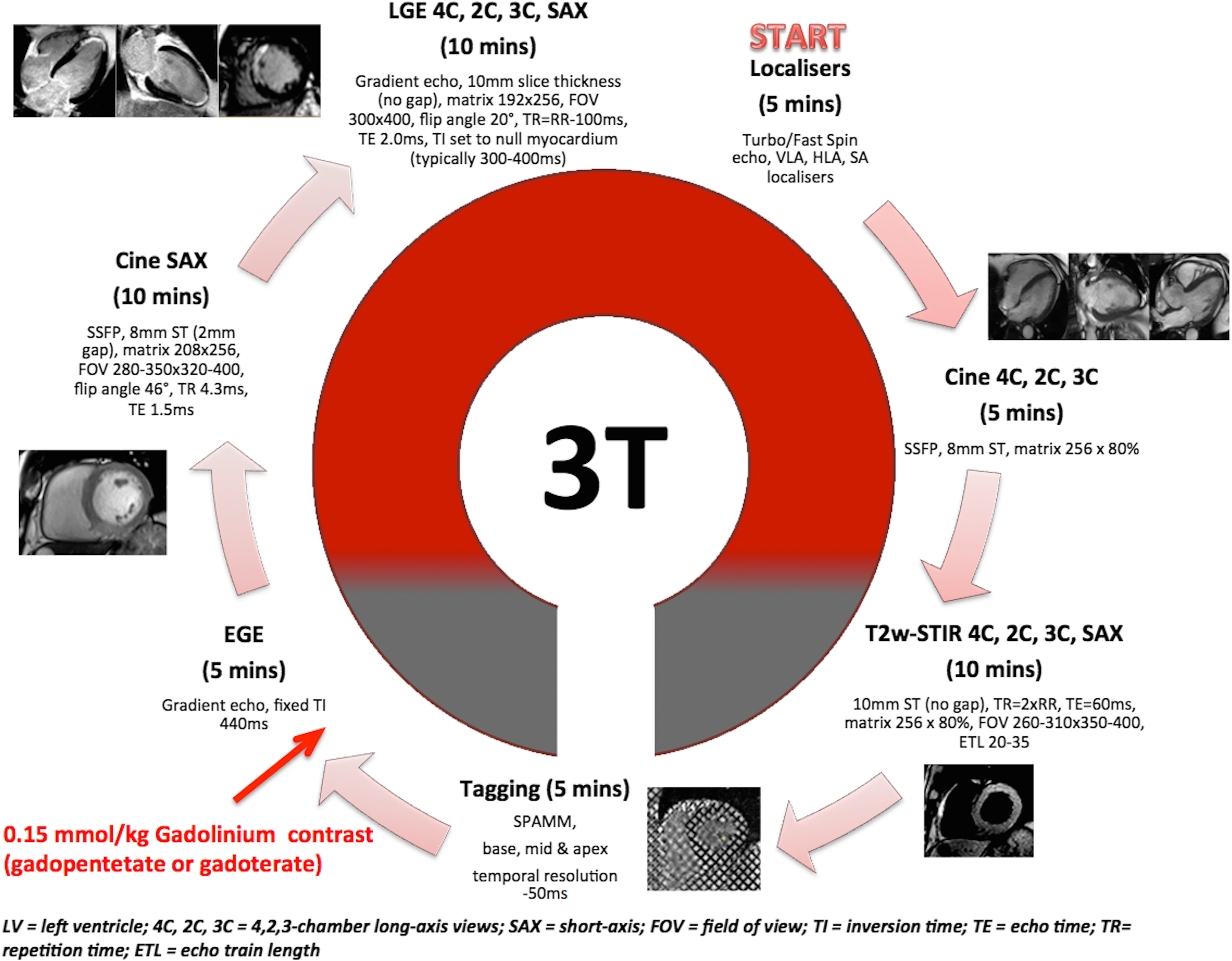

Supplement: Supplementary file 3 — Authors’ original file for figure 2 [file 13063_2014_2242_MOESM3_ESM.tif]
